# Supplementary material for: The causal role of circulating inflammatory markers in osteoporosis: a bidirectional Mendelian randomized study
Source: Front Immunol. 2024 Jul 18;15:1412298. doi: 10.3389/fimmu.2024.1412298 (PMC11291241; doi:10.3389/fimmu.2024.1412298)
Supplement: Supplementary file 1 [file DataSheet_1.zip › supplementary material/OSTEOPOROSIS-CIM/GCST90274777.tsv.gz_leave_one_out.pdf]

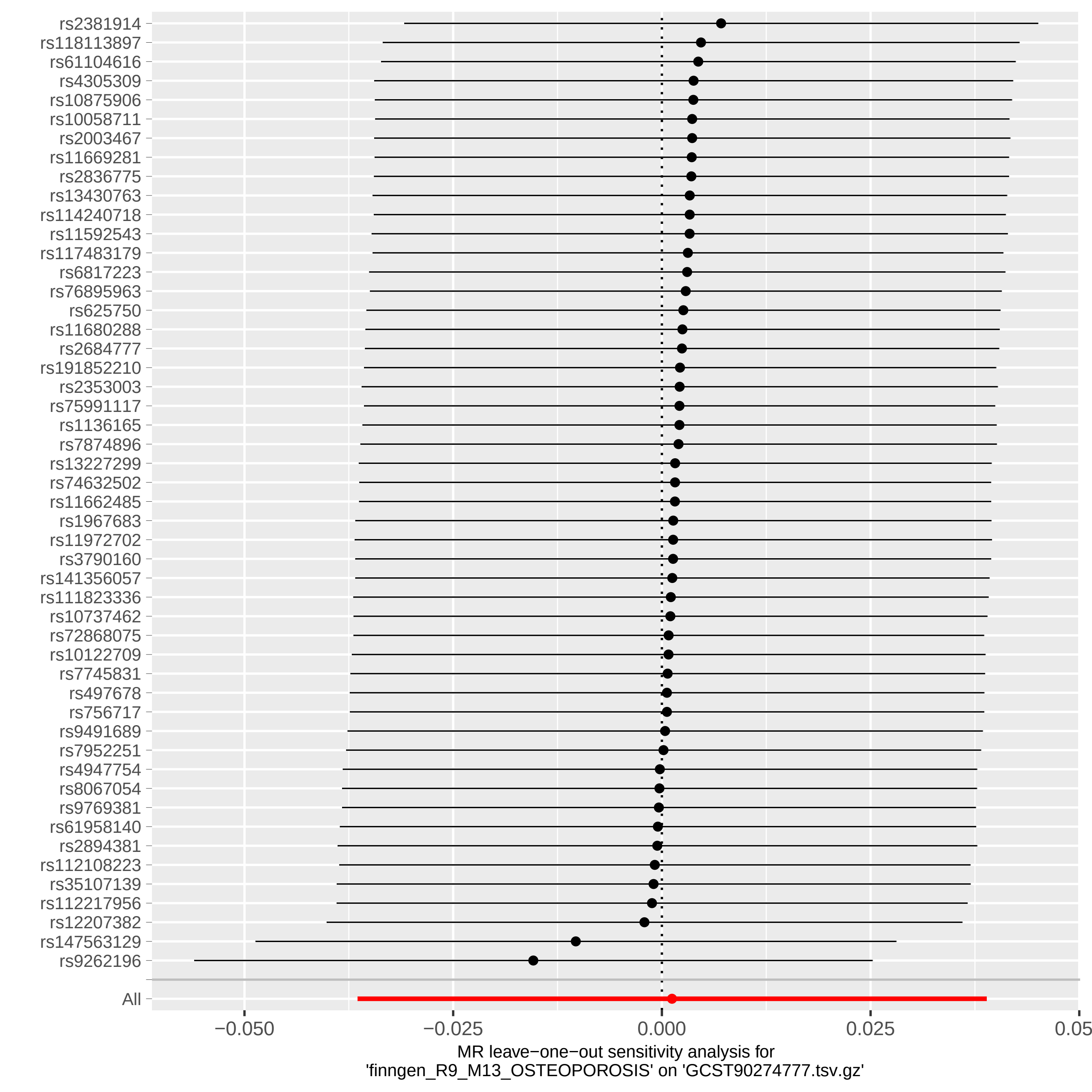
rs2381914
rs118113897
rs61104616
rs4305309
rs10875906
rs10058711
rs2003467
rs11669281
rs2836775
rs13430763
rs114240718
rs11592543
rs117483179
rs6817223
rs76895963
rs625750
rs11680288
rs2684777
rs191852210
rs2353003
rs75991117
rs1136165
rs7874896
rs13227299
rs74632502
rs11662485
rs1967683
rs11972702
rs3790160
rs141356057
rs111823336
rs10737462
rs72868075
rs10122709
rs7745831
rs497678
rs756717
rs9491689
rs7952251
rs4947754
rs8067054
rs9769381
rs61958140
rs2894381
rs112108223
rs35107139
rs112217956
rs12207382
rs147563129
rs9262196
All
−0.050
−0.025
0.000
0.025
0.05
MR leave−one−out sensitivity analysis for
'finngen_R9_M13_OSTEOPOROSIS' on 'GCST90274777.tsv.gz'
